# Exacerbated Headache-Related Pain in the Single Prolonged Stress Preclinical Model of Post-traumatic Stress Disorder

**DOI:** 10.1007/s10571-020-00962-8

**Published:** 2020-09-15

**Authors:** Yong Zhang, Kelly M. Standifer

**Affiliations:** 1 Department of Pharmaceutical Sciences, College of Pharmacy, University of Oklahoma Health Sciences Center, Oklahoma City, OK USA; 2 Oklahoma Center for Neuroscience, University of Oklahoma Health Sciences Center, Oklahoma City, OK USA

**Keywords:** PTSD, Chronic pain, Sodium nitroprusside, Nociceptin/orphanin FQ, Traumatic stress

## Abstract

Chronic headache pain is one of the most commonly reported comorbid pain conditions with post-traumatic stress disorder (PTSD) patients and resistant to effective treatment, yet no combined preclinical model of the two disorders has been reported. Here, we used a modified chronic headache pain model to investigate the contribution of single prolonged stress (SPS) model of PTSD with sodium nitroprusside (SNP)-induced hyperalgesia. Injection of SNP (2 mg/kg, i.p.) occurred every other day from day 7 to day 15 after initiation of SPS in rats. Paw withdrawal threshold (PWT) to von Frey stimuli and tail flick latencies (TFL) dramatically decreased as early as 7 days after SPS and lasted until at least day 21. Basal PWT and TFL also significantly decreased during the SNP treatment period. The lower nociceptive thresholds recovered in 6 days following the final SNP injection in SNP group, but not in SPS + SNP group. Elevated nociceptin/OFQ (N/OFQ) levels observed in cerebrospinal fluid of SPS rats were even higher in SPS + SNP group. Glial fibrillary acidic protein (GFAP) and N/OFQ peptide (NOP) receptor mRNA expression increased in dorsal root ganglia (DRG) 21 days after SPS exposure; mRNA increases in the SPS/SNP group was more pronounced than SPS or SNP alone. GFAP protein expression was upregulated in trigeminal ganglia by SPS. Our results indicate that traumatic stress exaggerated chronic SNP-induced nociceptive hypersensitivity, and that N/OFQ and activated satellite glia cells may play an important role in the interaction between both conditions.

## Introduction

Estimates of past-year and lifetime prevalence of PTSD are 4.7 and 6.1%, respectively, in the USA (Goldstein et al. 2016); this rate is much higher in those with chronic pain. For instance, prevalence in PTSD patients with chronic pain was 9.8% in the general population, and as high as 50.1% in veterans (Fishbain et al. 2017). In subgroup analysis, the PTSD prevalence was 20.5%, 11.2%, and 0.3% among persons with chronic widespread pain, headache, and back pain, respectively (Siqveland et al. 2017). PTSD comorbidity with chronic pain negatively influences the symptoms and course of treatment for both disorders (Sullivan et al. 2009; Rosenthal and Erickson 2013; Outcalt et al. 2015). The interaction of PTSD and chronic pain gained growing interest in last two decades; however, current knowledge relies almost entirely upon clinical observations; development of animal models of PTSD and chronic pain will help us better understand the underlying mechanisms contributing to this comorbidity.

Single prolonged stress (SPS), an established animal model for PTSD, simulates many of the PTSD symptoms reported in humans such as exaggerated negative feedback of the HPA axis, hypocortisolism (Zhang et al. 2012), enhanced fear and anxiety responses, and cognitive impairment (Yamamoto et al. 2009; Lisieski et al. 2018). We and others reported that SPS induces long-lasting mechanical and thermal allodynia and visceral and inflammatory hypersensitivity (Zhang et al. 2012, 2015; He et al. 2013). Clinical observations have long noted that nitroglycerin (NTG) evokes migraine-like headache pain (Demartini et al. 2019). NTG has been commonly used in rodent models of migraine wherein systemic administration of NTG produced acute hyperalgesia in rats (Tassorelli et al. 2003) and mice (Gölöncsér and Sperlágh 2014) lasting 2–4 h. Another nitric oxide donor, sodium nitroprusside, also produces allodynia and hyperalgesia in rats in similar time window (Galeotti and Ghelardini 2013). Recently, a new model of chronic migraine was introduced, in which 5 i.p. injections of NTG every other day over 9 days induced progressive and sustained hyperalgesia that took 7 days to recover after final NTG administration (Pradhan et al. 2014a, b). This model, if combined with SPS, would be promising to explore mechanisms underlying interaction of chronic pain and PTSD. However, prior to and during this study, it was difficult to find the more concentrated NTG needed for rat studies. Therefore, the current study modified the chronic headache pain model of Pradhan et al. (2014a, b) to utilize SNP to investigate if SPS affects severity and duration of headache-induced hyperalgesia, nociceptin/orphanin FQ (N/OFQ)-N/OFQ peptide receptor (NOP) levels, and astrocyte activation under both treatments. The results will help us to better understand how traumatic stress evoked changes noted with PTSD contribute to chronic pain to provide novel approaches for treatment of the two disorders.

## Methods

### Animals

Male Sprague–Dawley rats weighing 250–300 g were obtained from Charles River Labs (Wilmington, MA). After arrival, rats were acclimated to the animal facility for at least 7 days before experiments were initiated. They were housed in the animal facility under a 12-h light:12-h dark cycle (lights on at 0600 h) with free access to food and water. Experimental protocol was approved by the Institutional Animal Care and Use Committee of the University of Oklahoma Health Sciences Center. All experiments conformed to the guidelines of the International Association for the Study of Pain. Every effort was made to minimize animal discomfort and reduce the number of animals used. A total of 30 rats were used in this study.

### SPS and Drug Treatment

**Fig. 1:**
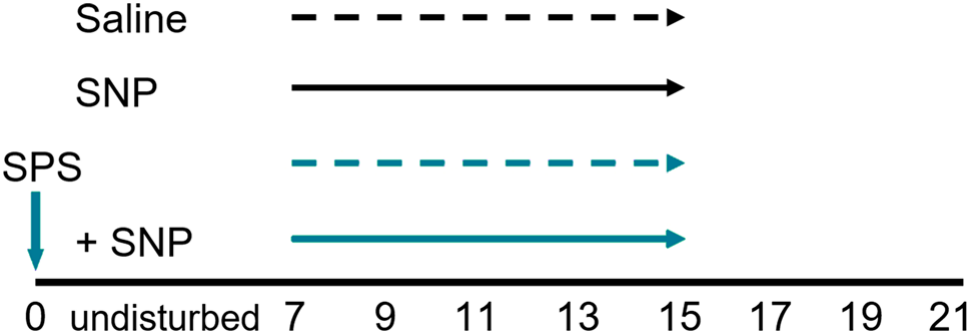
Experimental paradigm. Day 0 represents the beginning of the 7 day SPS period. SNP was administered (i.p. 2 mg/kg, QOD) to SNP or SPS + SNP groups from days 7 to day 15; control and SPS groups received the same volume of saline. Rats were assessed for baseline nociceptive sensitivity to mechanical and thermal stimuli as described above, prior to SPS initiation at day 0, and every other day from days 7 to 21. Rats were euthanized on day 21

Rats were randomly divided into control (*n* = 7), SNP (*n* = 8), SPS (*n* = 7), and SPS + SNP (*n* = 8) groups; the experimental paradigm is illustrated in Fig. 1. The SPS procedure proceeded as described (Zhang et al. 2012). After 7 days of acclimatization, rats were immobilized by placing inside a clear polyethylene restraint cone for 2 h, followed by grouped (3–4 rats) forced swimming for 20 min in a cylindrical Plexiglas tank (46 cm tall × 20 cm in diameter) filled with 22 °C water to a depth of 30 cm. Following 15 min of recovery and drying, animals were exposed to diethyl ether until loss of consciousness. Upon awakening, rats were returned to home cages and left undisturbed and alone for 7 days. From day 7 after SPS, sodium nitroprusside (SNP, Sigma-Aldrich, 2 mg/kg i.p.) was injected between 12 p.m. and 1 p.m. every other day (QOD) until day 15, for a total of 5 injections. Control and SPS rats received the same volume of saline vehicle. On day 21, animals were euthanized by i.p. injection of Beuthanasia (0.22 mg/kg, Henry Schein). Blood was withdrawn from the heart with an 18-gauge needle (between 13:00 and 15:00 h); it was maintained at room temperature for 30 min. Blood samples were centrifuged at 5000 × *g* at 4 °C for 5 min and the serum was collected and stored at − 80 °C. CSF from each rat was withdrawn by inserting a 26-gauge needle into the cysterna magna and immediately stored at − 80 °C. Trigeminal and L4–6 dorsal root ganglia were excised and frozen for biochemical analyses.

### Nociception Assessment

Experiments were performed in a climate-controlled room. Rats were acclimated in clear plastic boxes with a wire mesh floor for ~ 15 min, before an electronic von Frey filament (IITC Life Science, Inc., Woodland Hills, CA) was applied to the midplantar aspect of the left paw. Paw withdrawal threshold (PWT) was determined by averaging 3 assessments, spaced 3 min apart. Following PWT assessment, a tail flick unit with a tail temperature sensor (IITC Life Science, Inc., Woodland Hills, CA) was used to assess the nociceptive sensitivity by radiant heat tail flick latency (TFL) assay, with the lamp set at 25% active intensity (Burke et al. 2010). Rats were covered with a soft cloth and lightly held in place with the tail extended under the lamp. A radiant light source was focused 3 cm from the distal end of the tail. The lamp in the tail flick unit turned off as soon as the rat flicked its tail and the time lapse between the onset of irradiation; the flick of the tail was noted as TFL. Values from three measurements with 3 min intervals were averaged. Tail temperature was monitored by tail temperature probe for every test, and a cut-off limit of 12 s was set to prevent any tissue damage. Tail temperatures ranged between 22.5 and 23 °C. In addition, to nociceptive threshold testing, overall behavior was observed with special attention to any reduction in overall or hind limb movements.

Assessment of sensitivity to thermal and mechanical nociceptive stimuli was made prior to initiation of SPS and between 8 a.m. and 12 p.m. every other day from day 7 to 21 post SPS. Rats in control and SNP groups were assessed for PWT and TFL prior to and also 1, 2, and 4 h after SNP injection on day 7 to examine acute effects of SNP.

### Radioimmunoassay

After administration of Beuthanasia, N/OFQ levels in CSF and serum were determined by RIA kit (Phoenix Pharmaceuticals, CA) according to the manufacturer’s protocol, and data are presented as N/OFQ-IR. The sensitivity of the assay was < 10 pg/mL; non-specific binding was 2.9%. There was no cross-reactivity with Dynorphin A (1–17), enkephalin, or β-endorphin. Corticosterone levels in serum also were determined by RIA kit (MP Biomedicals, NY) according to the manufacturer’s manual. The sensitivity of the assay was 25 ng/mL and non-specific binding was 2.6%. Total amount of corticosterone was calculated and expressed as ng/mL. Samples and standards were assayed in duplicate. RIA curves and data were analyzed using GraphPad Prism 8.2 software.

### Real-Time PCR

TRI reagent (Sigma-Aldrich, MO) was immediately added to TG and DRG tissue for mRNA extraction. cDNA was synthesized using Super-Script III Reverse Transcriptase (Sigma-Aldrich, MO). Real-time PCR was performed using SYBR Green Master Mix (AnaSpec, Fremont, CA) and 125 nM forward and reverse primers (rat GFAP Fwd: 5′-CCT TGA GTC CTT GCG CGG CA-3′, Rev: 5′-TTG GCC CTC CTC CTC CAG CC-3′; rat GAPDH Fwd: 5′-ACCCAGAAGACTGTGGATGG-3′, Rev: 5′-CAC ATT GGG GGT AGG AAC AC-3′; rat NOP Fwd: 5′-GTT CAA GGA CTG GGT GTT CAG CCA GGT AGT-3′; rat NOP Rev: 5′-TGC TGG CCG TGG TAC TGT CTC AGA ACT CTT-3′; rat preproN/OFQ Fwd: 5′-TGC ACC AGA ATG GTA ATG TG-3′, Rev: 5′-TAG CAA CAG GAT TGT GGT GA-3′, all from Sigma-Aldrich) in an ABI 7000 Sequence Detection System (Applied Biosystems, CA). The GAPDH gene was used as an internal standard to which expression of other genes was normalized. Data were analyzed using the comparative *C*_t_ method, and compared with control values (Schmittgen and Livak 2008).

### Immunoblotting

DRG and TG tissue was homogenized with RIPA buffer (1% NP40, 0.5% Na_2_deoxycholate, 0.1% SDS, 5 mM EDTA, 10 mM NaF, PBS) containing freshly added protease and phosphatase inhibitors and incubated for 30 min on ice, with subsequent centrifugation at 14,000 × *g* for 10 min. After protein concentration determination with Pierce BCA protein assay kit (ThermoFisher), homogenates were solubilized with 4 × Laemmli buffer and stored at − 80 °C. Samples were resolved using SDS-PAGE on 8–15% Tris–glycine gels (∼20 μg total protein per well), transferred to polyvinylidene fluoride membranes, blocked with 5% milk in TBS-Tween buffer, and incubated overnight at 4 °C with goat anti-GFAP antibody (1:2000; RB-087A, ThermoFisher). Secondary antibody conjugated to horseradish peroxidase was incubated for 1 h at room temperature in 5% milk in TBS-T. Immunoreactive bands were visualized by chemiluminescence, captured with the Ultralum Omega Imaging System and densitometry was performed using Ultra Quant 6.0. Membranes were rinsed, stripped, and re-probed with anti-actin (1:2000; Sigma-Aldrich) as an internal loading control.

### Data Analysis

Symbols and error bars represent mean ± SD, respectively, unless otherwise stated. Statistical comparisons of behavioral and neurochemical data were performed by unpaired Student *t*-test or two-way ANOVA followed by Bonferroni’s post hoc analysis using GraphPad Prism 8.2 software. Results were considered statistically significant if *P* < 0.05.

## Results

**Fig. 2:**
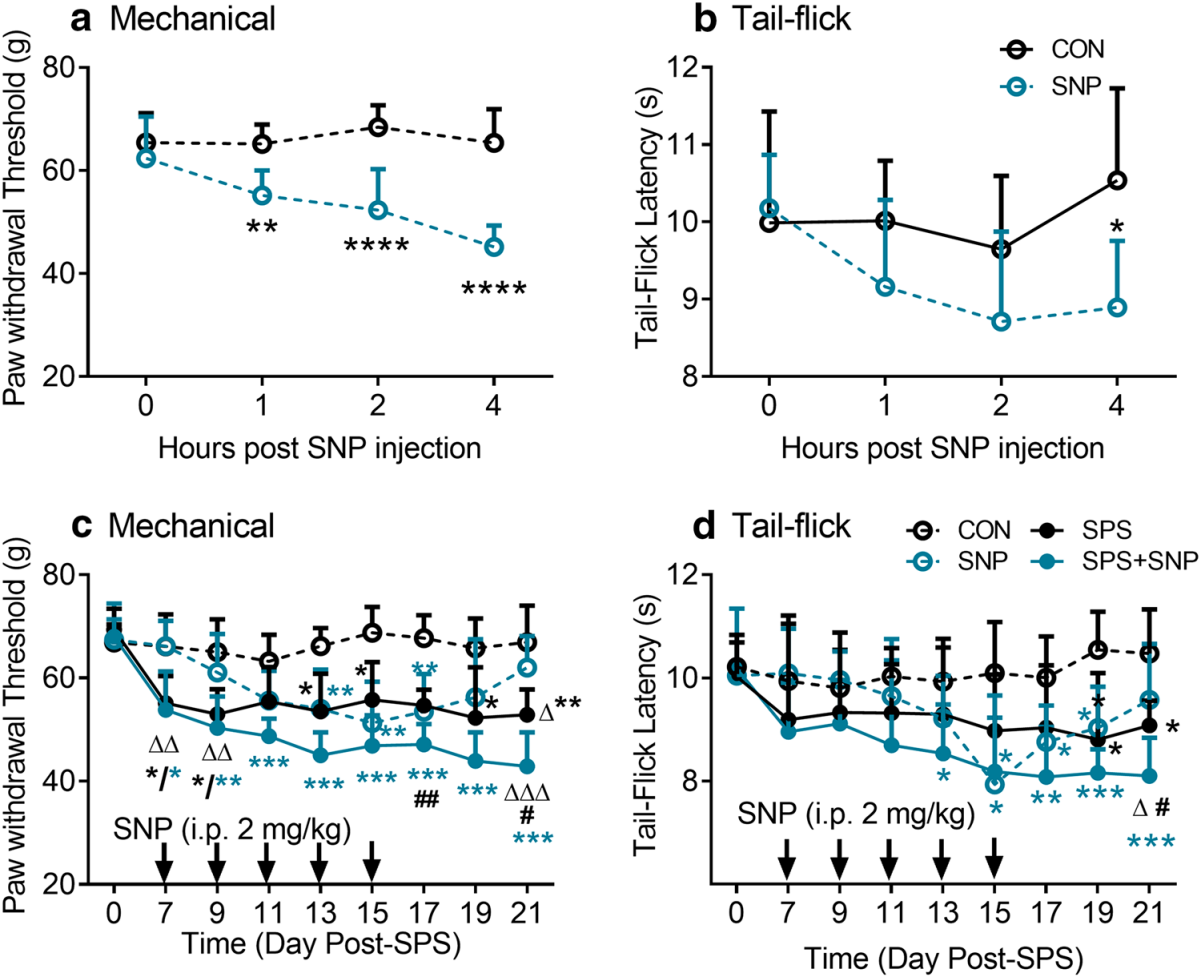
SNP + SPS increases mechanical and thermal nociceptive sensitivity more than either condition alone. Mechanical (**a**) and thermal (**b**) sensitivity were assessed before and 1, 2, 4 h after SNP injection in day 7 control and SNP groups. PWT decreased more quickly with SNP than TFL, but both were down by 4 h. Basal mechanical (**c**) and thermal (**d**) sensitivity were assessed at day 0 and every other day from day 7 to 21 before vehicle or SNP injection in all four groups. Basal PWT and TFL decreased by day 15 after SNP injection then recovered to control level by day 21. Decreased PWT exhibited as early as day 7 and maintained at least until day 21 after SPS. SPS + SNP further decreased PWT and TFL when compare to SPS or SNP alone. Data from **c** and **d** were analyzed using a mixed-effects model followed by Bonferroni’s post hoc analysis (*P < 0.05, **P < 0.01, SNP or SPS + SNP vs. VEH; ^Δ^*P* < 0.05, ^ΔΔ^*P* < 0.01, SPS + SNP vs SNP; ^#^*P* < 0.05, ^##^*P* < 0.01, SPS + SNP vs SPS)

Since nitric oxide donors nitroglycerin and sodium nitroprusside (SNP) induce allodynia and hyperalgesia to thermal stimuli, the ability of SNP (Galeotti and Ghelardini 2013) to produce acute hyperalgesia in rats was tested using assessment methods utilized in the headache (tail flick latency, TFL) and in the SPS literature (paw withdrawal threshold, PWT). PWT assessed centrally mediated responses to mechanical stimuli and TFL assessed spinal responses to thermal stimuli. PWT and TFL were determined over 4 h after SNP administration, as illustrated in Fig. 2a, b. Two-way, repeated measures ANOVA revealed significant effects of SNP treatment [*F*(1, 13) = 87.49, *P* < 0.0001], time [*F*(3, 39) = 4.873, *P* = 0.0057], and the interaction of SNP × Time [*F*(3, 39) = 5.406, *P* = 0.0033] on PWT with control and SNP rats (Fig. 2a). Bonferroni’s multiple comparisons post hoc tests revealed significant differences between control and SNP groups at 1, 2, and 4 h after SPS injection, indicating that SNP acutely induced mechanical allodynia over the 4 h period. Changes in thermal sensitivity were more subtle (Fig. 2b). Two-way ANOVA with repeated measures revealed that only SNP treatment produced a significant effect on TFL [*F*(1, 13) = 6.448, *P* = 0.0247], and only at 4 h post SNP injection (*P* < 0.05). These results suggest that the single injection of SNP introduced acute mechanical allodynia and mild thermal hyperalgesia that were still present after 4 h.

Once acute headache allodynia and hyperalgesia results were obtained with SNP, SNP was substituted for NTG (Pradhan et al. 2014a, b), a modified chronic headache model in rats. SNP (or vehicle) was administered every other day (QOD) between day 7 and day 15 post SPS (5 total injections), to control and SPS-treated rats. Sensitivity to mechanical and thermal stimuli was assessed before each injection that occurred between days 9 and 17 and continued until day 21, as shown in Fig. 2c, d (black arrows). Since each nociceptive assessment was made more than 42 h after the previous SNP injection, the hyperalgesia noted could not be attributed to an acute hyperalgesic effect of the prior SNP injection. Significant effects of treatment group: [*F*(3, 26) = 40.33, *P* < 0.001], time: [*F*(5.097, 126.2) = 9.352, *P* < 0.001], and treatment × Time: [*F*(24, 198) = 3.013; *P* < 0.001] on PWT were revealed by two-way ANOVA mixed-effects model (REML). Tukey’s multiple comparisons tests confirmed that SPS produced mechanical allodynia through day 21 as previously reported compared to control (*). SNP alone did not reduce PWT until day 13; and returned to baseline levels by day 21. PWT was significantly reduced in SPS + SNP rats compared to control (*), SNP alone (Δ), or SPS alone (#), that was maintained through day 21. Similar to PWT but less pronounced, the mixed-effects model found significant effects of treatment [*F*(3, 26) = 7.646, *P* = 0.0008], Time [*F*(5.902, 146.1) = 3.53; *P* = 0.0029], and significant interaction between treatment and time [*F*(24, 198) = 1.595, *P* = 0.0449] on TFL. Post hoc tests confirmed that SNP + SPS exposure produced significantly greater nociceptive sensitivity on day 21 than either SNP or SPS alone. However, while SNP treatment reduced TFL by day 13, this hypersensitivity was gone by day 21. Unlike responses to mechanical stimuli, SPS exposure did not reduce TFL significantly until day 19 and through day 21. Enhanced sensitivity to mechanical and thermal stimuli was found in the combined SPS + SNP group 4 days after the final SNP injection. Overall, these results suggest that SPS exacerbated SNP-induced hyperalgesia and prevented its recovery by day 21.

**Fig. 3:**
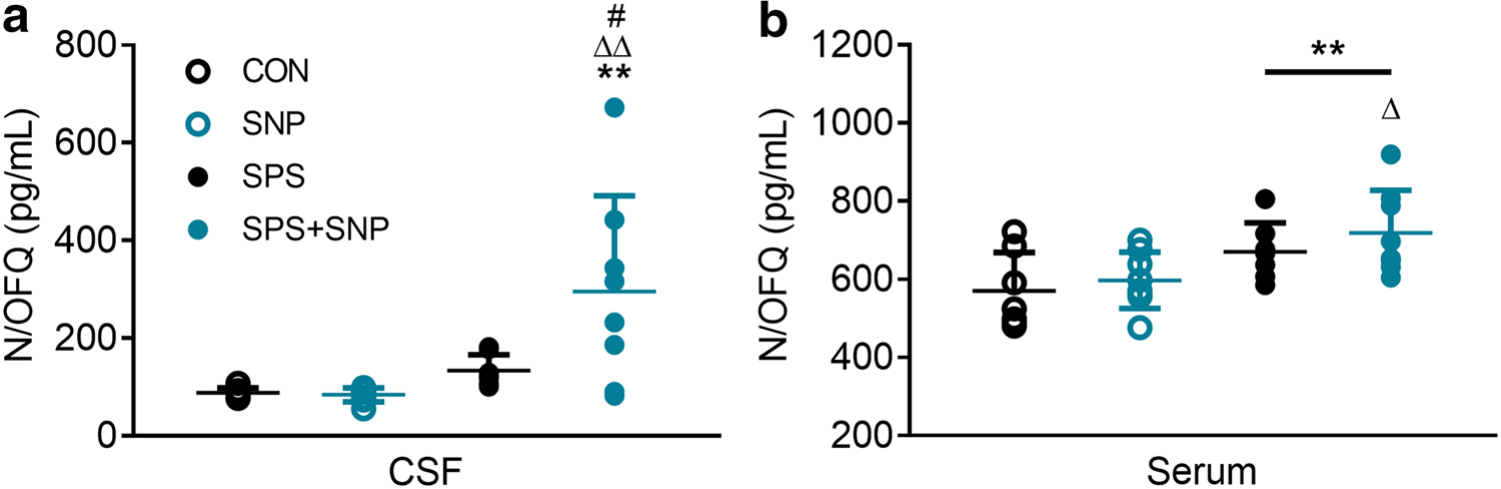
SNP + SPS treatment increased N/OFQ levels in CSF (**a**) and serum (**b**) more than either group alone. CSF N/OFQ levels increased with SPS alone at day 21; even more so in SPS + SNP group. Two-way ANOVA revealed significant interaction between SPS and SNP and significant effects of both treatments on N/OFQ level. Significant effect of SPS on N/OFQ levels was noted in serum as well. Data were analyzed by two-way ANOVA followed by Bonferroni’s post hoc analysis (*P < 0.05, **P < 0.01, SPS + SNP vs. VEH; ^ΔΔ^*P* < 0.01, SPS + SNP vs SNP; ^#^*P* < 0.05, SPS + SNP vs SPS)

We previously reported that SPS elevated N/OFQ levels in CSF and serum (Zhang et al. 2012, 2015) and were curious to see how the presence of SNP affected N/OFQ levels. Two-way ANOVA revealed a significant interaction between SPS and SNP [*F*(1, 26) = 4.82, *P* = 0.0372], as well as significant effects of SPS [*F*(1, 26) = 11.56, *P* = 0.0022] and SNP [*F*(1, 26) = 4.343, *P* = 0.047] on N/OFQ levels in CSF (Fig. 3a). Post hoc analysis indicated SPS + SNP treatment increased N/OFQ compared to all other groups. As reported previously, there was significant effect of SPS on serum N/OFQ [*F*(1, 26) = 11.33, *P* = 0.0024, Fig. 3b], with N/OFQ levels higher in SPS + SNP rats than controls.

**Fig. 4:**
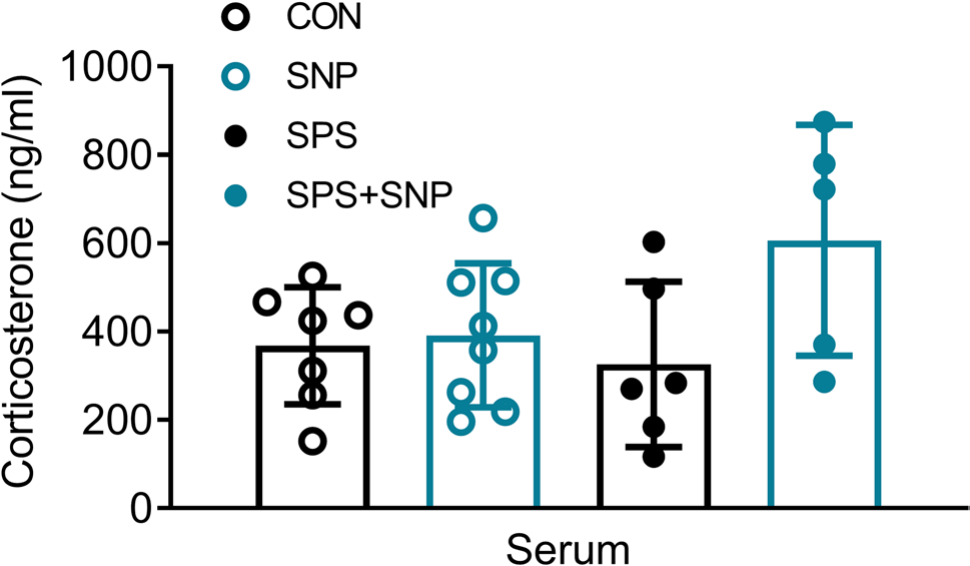
Effect of SPS and SNP treatment on serum corticosterone levels. Corticosterone levels in serum samples were determined by RIA at day 21 from control and SPS rats treated with or without SNP (i.p., 2 mg/kg QOD from days 7 to 15 after SPS; *n* = 7 in control, *n* = 8 in SNP, *n* = 6 in SPS, *n* = 5 in SPS + SNP). Serum corticosterone levels did not change in SPS or SNP rats, but increased in SPS + SNP rats. Data are plotted as means ± SD. *P < 0.05; two-way ANOVA followed by Bonferroni’s post hoc test

We previously reported that serum corticosterone levels remained unchanged at days 9 and 14 after SPS, but decreased at days 21 and 28 (Zhang et al. 2012). Two-way ANOVA revealed a significant effect of SNP on serum corticosterone [*F*(1, 22) = 4.351, *P* = 0.048] (Fig. 4), though no differences between groups were found.

**Fig. 5:**
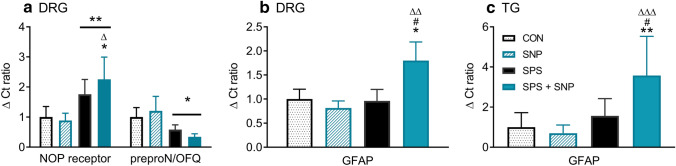
Effects of SNP and SPS on NOP receptor, preproN/OFQ ,and GFAP mRNA expression in DRG and TG. mRNA from L4 to L6 DRG and TG were extracted for real-time PCR. Target gene expression in SNP-, SPS-, and SPS + SNP-treated rats was normalized to control; error bars represent SEM. Two-way ANOVA analysis indicated significant effects of SPS on NOP and preproN/OFQ mRNA expression in DRG (**a**) and GFAP mRNA expression in DRG (**b**) and TG (**c**). GFAP changes in the SPS + SNP group was more pronounced than SPS alone. SNP treatment alone did not alter GFAP, NOP, or preproN/OFQ mRNA levels. Bonferroni’s post hoc analysis indicated specific differences (*P < 0.05, **P < 0.01, SPS + SNP vs. VEH; ^Δ^*P* < 0.05, ^ΔΔ^*P* < 0.01, SPS + SNP vs SNP; ^#^*P* < 0.05, SPS + SNP vs SPS)

**Fig. 6:**
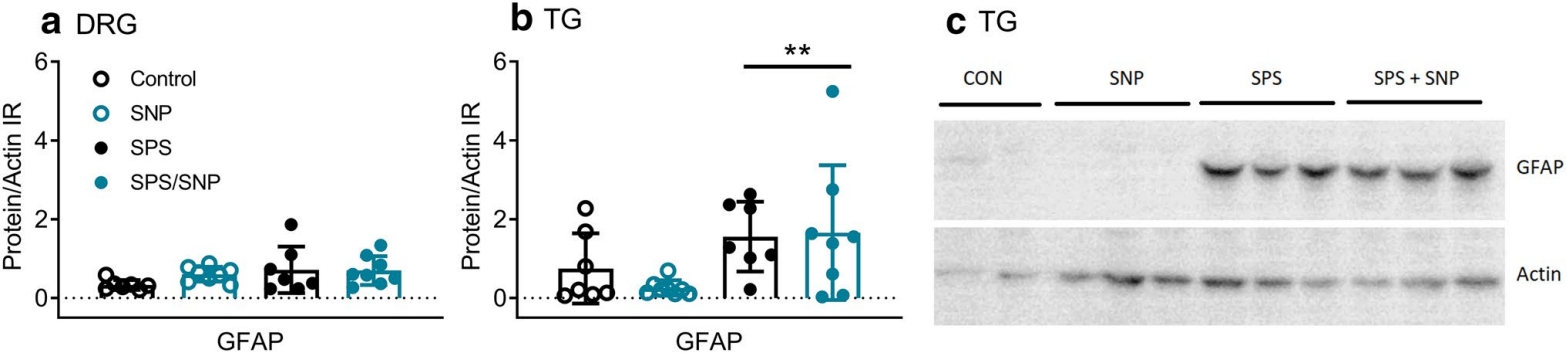
SPS increases GFAP protein expression in TG, but not DRG. Immunoblotting was performed using cell lysates from L4 to L6 DRG (**a**) and TG (**b**). Panel **c** contains representative immunoblots of GFAP in TG. GFAP expression was normalized to actin in all groups. Two-way ANOVA analysis indicated significant effect of SPS on GFAP expression in TG (**P < 0.01, **b**); post hoc analysis indicates that SPS + SNP was significantly higher than SNP alone (*P < 0.05)

The effect of SPS and/or SNP on the N/OFQ-NOP receptor system mRNA and GFAP mRNA and protein expression also were examined. TG and L4–6 DRG mRNA were prepared and subjected to real-time PCR analysis. As shown in Fig. 5a, two-way ANOVA revealed a significant effect of SPS on NOP receptor [*F*(1, 26) = 12.17, *P* = 0.0017] and preproN/OFQ [*F*(1, 26) = 6.844, *P* = 0.015] mRNA expression in DRG. NOP receptor mRNA expression in SPS + SNP rats was significantly higher than that in controls or SNP alone, as determined by Tukey’s multiple comparisons post hoc test. In contrast to its effects on the NOP receptor, the effect of SPS on preproN/OFQ mRNA was to decrease expression. There was significant interaction between SPS and SNP, and a significant effect of SPS on GFAP mRNA expression in DRG (Interaction: *F*(1, 26) = 6.764; *P* = 0.015; SPS: *F*(1, 26) = 5.796, *P* = 0.024) and TG (Interaction: *F*(1, 26) = 6.036; *P* = 0.021; SPS: *F*(1, 26) = 13.36, *P* = 0.001) as determined by two-way ANOVA. Moreover, post hoc analysis indicated significantly higher GFAP mRNA expression in SPS + SNP rats than in control, SPS, or SNP alone (Fig. 5b, c). To determine if GFAP protein levels increased, cell lysates from DRG and TG were prepared for immunoblotting. Though no significant interaction or effects of SPS or SNP were found for GFAP from DRG samples (Fig. 6a), there was a significant effect of SPS on TG GFAP, as determined by two-way ANOVA: [*F*(1, 26) = 7.849, *P* = 0.0095] (Fig. 6b, c).

## Discussion

Single prolonged stress (SPS) is an established model that mimics many physiological and behavioral alterations described in PTSD patients including enhanced negative feedback to the HPA axis, anxiety behavior, and cognitive impairments (see review, Yamamoto et al. 2009; Lisieski et al. 2018). Moreover, SPS induces persistent mechanical allodynia, thermal hyperalgesia (Zhang et al. 2012, 2015), and increased visceral hyperalgesia (He et al. 2013). Nitric oxide donors, such as nitroglycerin and SNP, have been used to recapitulate headache in human (Demartini et al. 2019; Guo et al. 2013) and rodent models of migraine (Moye and Pradhan 2017; Galeotti and Ghelardini 2013). Pain in rodents was reported as allodynia to cold plate test and hyperalgesia using a hot plate test. Repetitive injections of NTG produced sustained hyperalgesia using a TFL test (Pradhan et al. 2014a, b). That model of chronic migraine was not difficult to pair with SPS to investigate comorbid PTSD and chronic pain. Due to a nationwide shortage of nitroglycerin of sufficient concentration for use in rats during the time of this study, the second commonly used nitric oxide donor, SNP, was substituted for NTG in the chronic pain model. A single i.p. injection of SNP induced allodynia to mechanical stimuli and hyperalgesia to radiant heat in rats that lasted atleast 4 h. This is consistent with a previous study that showed hyperalgesia for 2 to 4 h that disappeared by 6 h after acute administration (Galeotti and Ghelardini 2013). The chronic pain model employed repetitive injection of SNP in the same paradigm as previously reported with NTG (Pradhan et al. 2014a, b). Again, rat PWT and TFL progressively decreased and with SNP and remained decreased until day 19, 4 days after the final treatment, with full recovery at day 21. This SNP effect is similar to NTG in mice (Pradhan et al. 2014a, b). Therefore, SNP appears to be a valuable substitute for NTG for a chronic migraine rat model.

Previous studies with SPS demonstrated that nociceptive hypersensitivity emerged as early as 7 days after initiation of SPS, and lasted at least 28 days (Zhang et al. 2012, 2015), and PWT results in this study were similar. The decreased TFL after SPS exposure was less profound than PWT, as TFL reductions were *n* until day 19. In contrast, we previously observed thermal hyperalgesia in the paw from day 7 through day 28 post SPS (Zhang et al. 2012). The difference between thermal sensitivity assessed in the paw versus the tail likely reflects TFL is a spinal nociceptive reflex, while paw withdrawal responses following SPS include central contributions from cortical and limbic structures. The radiant heat source intensity was reduced to 25% so that baseline TFL in control rats was increased to allow for reduced TFL with hyperalgesia resulting from SNP, SPS, or SPS + SNP. When combined with SNP treatment, SNP + SPS exposure produced significantly greater nociceptive sensitivity to both mechanical and thermal stimuli on day 21 than either SNP or SPS alone. A recent study demonstrated that 2 h of restraint stress for 3 consecutive days induced facial mechanical hypersensitivity in mice that was resolved by 14 days post stress. Following returning to baseline, a single low dose of SNP (i.p. 0.1 mg/kg) elicited mechanical hypersensitivity in stressed, but not in control animals, demonstrating the presence of migraine-like state after stress (Avona et al. 2020). This result and ours indicate that stressed animals are more vulnerable to SNP-induced acute and chronic hyperalgesia that is in line with clinical findings that many PTSD patients develop chronic pain. To our knowledge, the current study is the first to combine preclinical PTSD and chronic pain models to examine the contribution of each disorder to the other.

N/OFQ and NOP receptors are widely expressed in the CNS and involved in stress, anxiety, and pain processing (Zaveri 2016). In humans, N/OFQ levels were significantly increased in serum and CSF of patients with chronic pain (Ko et al. 2002; Raffaeli et al. 2006). However, in migraine patients, the plasma N/OFQ levels were reduced (Ertsey et al. 2005) or unchanged (Munksgaard et al. 2019). NOP receptor agonist Ro 64-6198 blocked cute NTG-induced hyperalgesia in mice (Targowska-Duda et al. 2020). We reported that SPS exposure increased N/OFQ in CSF and serum (Zhang et al. 2012), and that NOP receptor antagonist treatment reversed SPS-induced hyperalgesia (Zhang et al. 2015), consistent with an important role of the N/OFQ-NOP receptor system in the development and maintenance of pain hypersensitivity after traumatic stress. In this current study, repetitive administration of SNP did not alter N/OFQ levels in CSF and serum. SPS increased N/OFQ level in CSF, but SPS + SNP further increased N/OFQ levels in both CSF and serum higher than SPS alone, suggesting that N/OFQ is associated with the enhanced hyperalgesia noted in the presence of a traumatic stressor. Future studies will explore this relationship further.

N/OFQ and NOP present in lumbar dorsal horn and DRG, mainly small- and medium-sized neurons (Chen and Sommer 2006; Ozawa et al. 2018) that are important for the regulation of acute thermal and mechanical pain, and injury-induced hyperalgesia. A similar pattern of distribution exists for N/OFQ precursor preproN/OFQ mRNA (Harrison and Grandy 2000; Mogil and Pasternak 2001; Mika et al. 2003). NOP and ppN/OFQ mRNA were upregulated in inflammatory (Itoh et al. 2001) and neuropathic (Briscini et al. 2002; Pettersson et al. 2002; Mika et al. 2003) pain models. At the protein level, both N/OFQ and NOP immunoreactivity were upregulated after nerve injury and inflammation (Chen and Sommer 2006); upregulation of NOP in periaqueductal gray (PAG) and its mRNA in PAG and amygdala is found with SPS (Zhang et al. 2015). Here, we examined N/OFQ and NOP receptor mRNA at the level of the primary afferent neuron cell bodies. SNP treatment did not alter NOP or ppN/OFQ mRNA levels in DRG 6 days after the final treatment, when pain threshold had returned to baseline. NOP mRNA expression increased after SPS, and was even higher in SPS + SNP rats, suggesting that NOP biosynthesis is upregulated in DRG similar to its ligand, N/OFQ, in CSF. Contrary to upregulation of N/OFQ levels in CSF, ppN/OFQ mRNA expression decreased in DRG after SPS, and was further suppressed when SPS was combined with SNP. N/OFQ is produced by neuronal, glial and immune cells (Lambert 2008). Decreased ppN/OFQ mRNA level in DRG may indicated that DRG is not the source of increased N/OFQ content in CSF, or that ppN/OFQ transcription and translation are differentially regulated. It is common to find that recently translated mRNA levels decreased relative to their resulting protein product.

PTSD is associated with long-term dysregulation of the hypothalamus–pituitary–adrenal axis (HPA) axis; cortisol levels are increased in some PTSD patients and decreased in others (Handwerger 2009). Migraine patients showed higher variation of cortisol levels (Ziegler et al. 1979). NTG generated more cortisol release in migraineurs, however, that cortisol release did not correlated with headache pain (Lippi and Mattiuzzi 2017; Baksa et al. 2019). Elevated serum CORT levels were observed after a single dose of NTG (Farajdokht et al. 2016), and repetitive NTG injections induced chronic migraine when detected 2 days after final treatment (Farajdokht et al. 2017). In our study, SNP did not alter serum CORT level 6 days after final injection. This discrepancy is likely due to different time points of blood sample collection. In the SPS model, circulating CORT levels remained unchanged before day 14 and decreased 28 days post SPS (Zhang et al. 2012). We also did not see serum CORT level change at day 21 after SPS exposure. However, the SPS + SNP group exhibited elevated serum CORT level than other groups, it seems that SPS prolonged the effect of SNP to activate the HPA axis resulting in elevation of adrenocorticotrophic hormone (ACTH) and serum CORT levels.

Growing evidence indicates that satellite glia cells (SGCs) in sensory ganglia play important roles in pain modulation. SGCs are activated after nerve injury (Liu et al. 2012; Zhang et al. 2009) and inflammatory pain (Takeda et al. 2007, 2009) in TG and DRG, and play an active role in the development of persistent pain. Several studies suggested that PTSD-like conditions induced astrocytic inhibition in CNS, specifically in hippocampus and frontal cortex (Perez-Urrutia et al. 2017; Saur et al. 2016; Han et al. 2015; Xia et al. 2013). The response of SGCs to SPS and chronic migraine has not been reported. Here we examined expression of GFAP, a marker of activated SGCs (Takeda et al. 2007), in TG and DRG, which is the first station in pain pathways. We found that SPS upregulated GFAP mRNA expression in TG and DRG, as well as protein levels in TG. Similar to the observations in pain assessment, SNP alone did not alter GFAP expression at the time when hyperalgesia recovered, but it accentuated the GFAP changes at messenger level by SPS when two treatments combined, indicating that SGC activation may contribute to prolonged hyperalgesia after SPS exposure. The absence of GFAP protein change in DRG is unclear. A recent study compared transcriptome and translatome activity in TG and DRG, and found that translational efficiency in mammalian target of rapamycin (mTOR)-related genes is higher in the TG compared with DRG, whereas other genes associated with the negative regulator of mTOR have higher translational efficiency in DRG (Megat et al. 2019). The distinct translational profiling may result in the discrepancy of GFAP protein expression in these two tissues.

## Conclusion

The novelty of this study is combining two preclinical models of PTSD and chronic migraine to explore how the combination alters responses to each condition. Our major findings are that SPS exacerbated severity and duration of chronic pain by SNP, accompanied by elevated N/OFQ levels in serum and CSF, higher expression of NOP mRNA and lower expression of ppN/OFQ mRNA in DRG, and enhanced expression of GFAP mRNA in TG and DRG. Taken together, our results suggest that the N/OFQ-NOP system and activated SGCs may play important roles in the interaction between PTSD-like and chronic pain conditions, presenting two potential targets for therapeutic approach of comorbid PTSD and pain.

## Data Availability

All of the data collected are reported herein.
